# High efficiency of alphaviral gene transfer in combination with 5-fluorouracil in a mouse mammary tumor model

**DOI:** 10.1186/1471-2407-14-460

**Published:** 2014-06-20

**Authors:** Anna Zajakina, Jelena Vasilevska, Dmitry Zhulenkovs, Dace Skrastina, Artjoms Spaks, Aiva Plotniece, Tatjana Kozlovska

**Affiliations:** 1 Department of Cell Biology, Biomedical Research and Study Centre, Ratsupites Str., 1, Riga LV-1067, Latvia; 2 P. Stradins Clinical University Hospital, Riga, Latvia; 3 Latvian Institute of Organic Synthesis, Riga, Latvia

**Keywords:** Semliki Forest virus, Cytotoxic effect, 5-fluorouracil, Combined cancer treatment, 4 T1 tumor

## Abstract

**Background:**

The combination of virotherapy and chemotherapy may enable efficient tumor regression that would be unachievable using either therapy alone. In this study, we investigated the efficiency of transgene delivery and the cytotoxic effects of alphaviral vector in combination with 5-fluorouracil (5-FU) in a mouse mammary tumor model (4 T1).

**Methods:**

Replication-deficient Semliki Forest virus (SFV) vectors carrying genes encoding fluorescent proteins were used to infect 4 T1 cell cultures treated with different doses of 5-FU. The efficiency of infection was monitored via fluorescence microscopy and quantified by fluorometry. The cytotoxicity of the combined treatment with 5-FU and alphaviral vector was measured using an MTT-based cell viability assay. *In vivo* experiments were performed in a subcutaneous 4 T1 mouse mammary tumor model with different 5-FU doses and an SFV vector encoding firefly luciferase.

**Results:**

Infection of 4 T1 cells with SFV prior to 5-FU treatment did not produce a synergistic anti-proliferative effect. An alternative treatment strategy, in which 5-FU was used prior to virus infection, strongly inhibited SFV expression. Nevertheless, *in vivo* experiments showed a significant enhancement in SFV-driven transgene (luciferase) expression upon intratumoral and intraperitoneal vector administration in 4 T1 tumor-bearing mice pretreated with 5-FU: here, we observed a positive correlation between 5-FU dose and the level of luciferase expression.

**Conclusions:**

Although 5-FU inhibited SFV-mediated transgene expression in 4 T1 cells *in vitro*, application of the drug in a mouse model revealed a significant enhancement of intratumoral transgene synthesis compared with 5-FU untreated mice. These results may have implications for efficient transgene delivery and the development of potent cancer treatment strategies using alphaviral vectors and 5-FU.

## Background

Several preclinical studies in recent years have demonstrated therapeutic synergy between viral vectors and chemotherapy [1,2]. As reported previously, chemical compounds might be acting as adjuvants for the applied genetic vaccines [3] and/or could enhance the infectivity and gene transfer efficiency of the viral vector [4]. Among the potential therapeutic viruses, alphaviral vectors are good candidates for cancer therapy because of the high level of transgene expression and their ability to mediate strong cytotoxic effects through the induction of p53-independent apoptosis [5,6]. The advantages of alphaviral vectors also include a low specific immune response against the vector itself, the absence of vector pre-immunity and a high level of biosafety [7,8].

Alphaviruses are enveloped viruses that belong to the *Togaviridae* family and contain a positive-strand RNA genome. The classic vectors for the expression of heterologous genes were developed primarily based on Semliki Forest virus (SFV) and Sindbis virus (SIN) replicons. In these vectors, a heterologous insert replaces the structural genes under the control of the 26S viral subgenomic promoter [9,10]. The vector RNA can be packaged into recombinant alphaviral particles in cells via co-transfection with a helper RNA encoding structural genes (capsid and envelope). Upon infection, the vector RNA replicates and generates a high level of expression of the heterologous gene. The vector cannot propagate because it lacks the genes encoding the required viral structural proteins. Replication of the recombinant alphaviral genome, which occurs on the cytoplasmic membrane, causes cellular apoptosis, even in the absence of viral structural gene expression [11].

Due to the rapid induction of apoptosis in infected cells, treatment with natural oncolytic alphaviral vectors results in tumor regression [12-15]. Administration of replication-deficient vectors encoding reporter or immunomodulator genes, such as cytokines or growth factors, has also been demonstrated. This leads to successful tumor inhibition or complete regression in animal models [16-19]. Nevertheless, the application of alphaviral immunogene therapy in a clinical study using Venezuelan equine encephalitis (VEE) virus (VEE/CEA) in phase I/II demonstrated insufficient anti-tumor efficacy in patients, most likely due to the inefficient induction of anti-tumor immune responses in patients with end-stage disease [20]. Moreover, the alphaviral vectors were administered to patients after standard treatment (usually chemotherapy), which may significantly reduce the efficiency of alphavirus infection and transgene expression. Remarkably, the majority of the successful preclinical studies using alphaviral vectors were performed in animal cancer models that did not involve pretreatment with chemical drugs. Therefore, the effect of combined chemotherapy and alphaviral therapy has not been comprehensively studied.

The efficacy of virotherapy depends on specific tumor targeting and the level of viral replication [21]. It has been reported that the application of classical chemical drugs, e.g., 5-fluorouracil (5-FU) and gemcitabine, in combination with oncolytic herpes or adenoviral vectors make cancer cells more prone to virus infection and replication [4,22], thereby enhancing the therapeutic effects of the viral vector. Alternatively, the viruses may improve the chemotherapy outcomes. For example, Newcastle disease virus has been shown to assist in overcoming cisplatin resistance in a lung cancer mouse model [23]. Moreover, the use of herpes simplex virus following doxorubicin treatment was demonstrated to eradicate chemoresistant cancer stem cells in a murine breast cancer model [24]. Also co-administration of reovirus with docetaxel synergistically enhanced chemotherapy in a human prostate cancer model [25], allowing reduced doses of chemotherapeutics to be used. Furthermore, the combination of an asymptomatic low dose of 5-FU with recombinant adenoviruses produces a synergistic effect in various cell lines and *in vivo* tumor models [26-30]. Although the detailed molecular mechanism underlying the therapeutic benefits of the combined treatment remains unknown, such a treatment has already demonstrated promising results in a clinical setting [31,32].

Whether the synergistic anti-tumor effect can be achieved using a drug combination that includes alphaviral vectors has been poorly investigated. One study showed that application of a Sindbis vector with oncolytic properties in combination with the topoisomerase inhibitor irinotecan in SCID mice bearing human ovarian cancer resulted in prolonged animal survival [33]. The authors highlight the role of natural killer cells in the induction of the anti-cancer effect by the combined treatment. Targeting of different anti-cancer mechanisms involving immune cell activation could lead to effective combinatorial therapies, though these would have to be evaluated in immunocompetent tumor models.

Using a 4 T1 mouse mammary tumor model, we investigated the efficiency of combined 5-FU and SFV vector treatment. We focused on the inhibition of cell proliferation and efficiency of transgene delivery under combined treatment *in vitro* and *in vivo*.

## Methods

### Cell lines and animals

BHK-21 (baby hamster kidney cells) and 4 T1 cells (metastasizing mammary carcinoma from BALB/c mice) were obtained from the American Type Culture Collection (ATCC/LGC Prochem, Boras, Sweden). BHK-21 cells were propagated in BHK - Glasgow MEM (GIBCO/Invitrogen, Paisley, UK) supplemented with 5% fetal bovine serum (FBS), 10% tryptose phosphate broth, 2 mM L-glutamine, 20 mM HEPES, streptomycin 100 mg ml^−1^ and penicillin 100 U ml^−1^. The 4 T1 cell line was cultured in Dulbecco’s minimal essential medium (GIBCO/Invitrogen) supplemented with 10% FBS, 2 mM L-glutamine, streptomycin 100 mg ml^−1^ and penicillin 100 U ml^−1^. Specific pathogen-free 4- to 6-week-old female BALB/c mice were obtained from Latvian Experimental Animal Laboratory of Riga Stradin’s University and maintained under pathogen-free conditions in accordance with the principles and guidelines of the Latvian and European Community laws. All experiments were approved by the local Animal Protection Ethical Committee of the Latvian Food and Veterinary Service (permission for animal experiments no. 32/23.12.2010).

### Production of SFV (SFV/EGFP, SFV/DS-Red, SFV/EnhLuc) and SIN (SIN/EGFP) recombinant virus particles

The pSFV1 [9] and pSinRep5 [10] vectors were used in this study. The enhanced green fluorescent protein (EGFP) gene was introduced into both vectors under the 26S subgenomic promoter. The EGFP gene was cut out of the pEGFP-C1 plasmid (Clontech, CA, USA) with *Nhe*I and *Hpa*I restriction endonucleases, treated with T4 DNA polymerase (Thermo Scientific, Lithuania) to blunt the DNA ends and ligated with the pSFV1 and pSinRep5 vectors, which were cleaved with *Sma*I and *Pml*I, respectively. Additionally, a pSFV1/DS-Red construct carrying the red fluorescent protein gene (DS-Red) [34] was generated. The DS-Red gene was amplified by PCR (primers: 5′-ATTAGGATCCACCGGTCGCCACCATG-3′ and 5′-TATCCCGGGCTACAGGAACAGGTGGTG-3′) using the pDsRed-Monomer-C1 plasmid as a template (Clontech, CA, USA). The PCR fragment was cleaved with *Bam*HI and *Sma*I and ligated into a pSFV1 vector cleaved with the same enzymes. An SFV vector carrying the firefly luciferase gene was used for the *in vivo* experiments [35].

The resulting plasmids were used to produce recombinant virus particles as previously described [35]. pSFV-Helper [9] and pSIN-DH-EB helper [10] were used to produce the SFV and SIN particles, respectively. The DNA template was removed by digestion with RNase-free DNase (Fermentas, Lithuania). The viral titers (infectious units per ml, iu ml^−1^) were quantified by infecting BHK-21 cells with serial dilutions of viral stock and analyzing EGFP or DS-Red expression via fluorescence microscopy on a Leica DM IL microscope (Leica Microsystems Wetzlar GmbH, Germany). For the *in vivo* application, SFV/EnhLuc viral particles (v.p.) were concentrated, and the viral titer was quantified by Real-time PCR as previously described [35].

### Infection of cell lines with recombinant virus particles

Cells were cultivated in 24-well plates at a density of 2 × 10^5^ cells per well in a humidified 5% CO_2_ incubator at 37°C. For transduction, the cells were washed twice with PBS containing Mg^2+^ and Ca^2+^ (Invitrogen, UK). Next, 0.3 ml of the solution containing the virus particles was added. The SFV/EGFP, SFV/DS-Red and SIN/EGFP virus particles were diluted in PBS (containing Mg^2+^ and Ca^2+^) to achieve a multiplicity of infection (MOI) of 10. The cells were incubated for 1 h in a humidified 5% CO_2_ incubator at 37°C. The control cells (uninfected) were incubated with PBS (containing Mg^2+^ and Ca^2+^). After incubation, the solution containing the virus was replaced with 0.5 ml of growth medium. The cells were gently washed with PBS and transferred to fresh medium every day.

### MTT cell proliferation assay

The cytotoxicity was quantified using the MTT (3-[4,5- dimethylthiazol-2-yl]-2,5-diphenyl tetrazolium bromide)-based cell viability assay. Cells were infected in 24-well plates as described above, and proliferation was analyzed 0, 1, 2, 3, 4 and 5 days after infection. The medium was replaced with 0.3 ml of solution containing 0.5 mg ml^−1^ MTT (Affymetrix, Cleveland, USA) dissolved in D-MEM without phenol red (GIBCO/Invitrogen, UK) supplemented with 5% FBS. The cells were incubated for 2 h in a humidified 5% CO_2_ incubator at 37°C. After incubation, the formazan crystals were dissolved by adding 0.3 ml of MTT solubilization solution consisting of 10% Triton X-100 and 0.1 N HCl in anhydrous isopropanol. The absorbance was measured using a microplate spectrophotometer (BioTek Instruments, Winooski, USA) at a test wavelength of 570 nm and a reference wavelength of 620 nm. Cell viability (%) was obtained using the following equation: Percent cell viability = (test 570 nm – 620 nm)/(control 570 nm – 620 nm) × 100, where the control is the value obtained from uninfected cells (the standard error of the control was less than 3% for days 0–3 and less than 6% for days 4–5 in three independent experiments).

### Fluorescence-activated cell sorting (FACS) analysis

Cells were infected on 6-well plates with SFV/EGFP and SIN/EGFP virus particles at an MOI of 10 as described above (1 ml of virus-containing solution was used for the infection). The infected cells were harvested 24 h after infection. Detached cells were harvested from the cell medium by centrifugation, and attached cells were trypsinized. The collected cells (approximately 10^6^) were washed with PBS and resuspended in 1 ml of PBS. For propidium iodide (PI) staining, the cells were incubated with 10 μl of 50 μg ml^−1^ PI solution (Becton Dickinson Biosciences, San Jose, California, USA) and immediately processed for FACS analysis. EGFP and PI fluorescence was measured using a FACSAria II (Becton Dickinson Biosciences, San Jose, California, USA). The FACS data were analyzed by BD FACSDiva 6.1.2 software. Uninfected cells were used as a negative control for both the PI and EGFP FACS analysis and contained approximately 1-2% PI-positive cells in 4 T1 culture.

### Fluorometry of infected/reinfected cells

Cells were seeded on 24-well plates and infected with SFV/EGFP as described above. After 24, 48 and 72 h, the infected cells were reinfected with the SFV/DS-Red virus. DS-Red fluorescence was measured 24 h after each reinfection using a fluorometric plate reader (Tecan Infinite M 200, Austria) with an excitation wavelength of 535 nm and an emission wavelength of 590 nm. The fluorometry data were expressed as the percentage of the reinfected cell fluorescence units relative to the fluorescence units obtained from the control cells infected with SFV/DS-Red alone (positive control, 100%). The experiments were performed in triplicate.

### Treatment of cells with 5-FU

5-FU powder (Sigma, St. Louis, MO, USA) was dissolved in DMSO at a concentration of 70 mg ml^−1^ and further diluted in filtered water to 7 mg ml^−1^. 4 T1 cells were seeded in a 24-well plate (2 × 10^5^ cells per well). The next day, the cells were treated with medium containing 5-FU at 13, 26, 65 or 130 μg ml^−1^. Every day for 5 days, the cells were gently washed with PBS to remove dead and detached cells, and fresh medium containing 5-FU was added. The control cells were not treated with 5-FU. The MTT cell proliferation assay was performed 0, 1, 2, 3, 4 and 5 days after the start of 5-FU treatment. The presence of DMSO traces did not affect 4 T1 cell proliferation.

### Induction of tumor nodules

A 4 T1 mouse mammary tumor model was established as previously described [35]. Briefly, 4 T1 tumor cells were resuspended in PBS at a final concentration of 2.5 × 10^6^ cells ml^−1^. Two hundred microliters of the 4 T1 cell suspension were subcutaneously injected above the right shoulder blade of the mice. After 10 days, the obtained tumor volumes reached at least 1000 mm^3^.

### 5-FU treatment and SFV/EnhLuc injection *in vivo*

5-FU powder (Sigma, St. Louis, MO, USA) was dissolved in DMSO at a concentration of 300 mg ml^−1^ and then diluted in filtered water to 30 mg ml^−1^. 4 T1 tumor-bearing mice (n ≥ 5) were treated with 5-FU at different doses (40, 150 or 400 mg kg^−1^) via peroral administration 4 times over a period of 8 days (every other day). One hour after the last 5-FU treatment, the mice were inoculated either i.t. (intratumoral) or i.p. (intraperitoneal) with 200 μl (4 injections of approximately 50 μl each) or 300 μl of SFV1/EnhLuc particle-containing stocks (6 × 10^9^ v.p. ml^−1^), respectively. As a control, 4 T1 tumor-bearing mice not treated with 5-FU were i.t or i.p. inoculated with the same dose and volume of SFV1/EnhLuc.

### Analysis of luciferase gene expression in mouse organs and tumors

The Luc gene expression level was estimated by measuring luciferase enzymatic activity in tissue homogenates 24 h after SFV/EnhLuc virus administration. The tumors and organs were excised and manually homogenized in a 1x concentration of ice-cold lysis buffer (Cell Culture Lysis buffer, Promega) containing a protease inhibitor cocktail (10 μl per 1 ml of lysis buffer) (Sigma, St. Louis, MO, USA). After homogenization, the samples were centrifuged for 10 min at 9000 × *g*, and the protein concentration was determined in tissue lysates using the BCA Protein Assay Kit (Pierce™ BCA Protein Assay Kit, Thermo Scientific, UK). Luciferase activity was measured by adding 100 μl of freshly reconstituted luciferase assay buffer to 20 μl of the tissue homogenate (Luciferase Assay System, Promega, USA) and then was quantified as relative light units (RLUs) using a luminometer (Luminoskan Ascent, Thermo Scientific, UK). The RLU values were expressed per mg of protein in the lysates. As a negative control, 4 T1 tumor-bearing mice were inoculated with PBS, and the maximal negative values were subtracted from the presented results.

The efficacy index of the 5-FU and SFV combined treatment was calculated using the formula (RLU in 5-FU treated mice/RLU in 5-FU non-treated mice)/(tumor weight in 5-FU treated mice/tumor weight in 5-FU non-treated mice). For example: the efficacy index = (3497925.0/1397062.5)/(681.3/690.9) = 2.5. The efficacy index thus reflects the level of SFV expression (increase in RLU) and the effect of the 5-FU treatment (reduction in tumor weight).

### Analysis of FITC-dextran accumulation

The first group of 4 T1 tumor-bearing mice (n = 3) was treated with 150 mg kg^−1^ 5-FU as described above and the second group (n = 3) was untreated with 5-FU. Next day after the last 5-FU treatment the mice from both groups were inoculated i.v. with 120 μl of FITC-dextran 2000 kDa solution (40 mg ml^−1^ in PBS) (Sigma). Two hours later tumors were collected and incubated overnight in 4% paraformaldehyde. After cryoprotection in 20% sucrose tumors were frozen in OCT compound (Sigma). Cryosections (10 μm) were prepared and the intensity of FITC-dextran leakage was visualized by fluorescent microscopy. Pixels of images were measured by ImageJ software.

### Analysis of IFN-alpha in tumor lysates

Two groups of 4 T1 tumor-bearing mice (n = 6 each) were either treated or non-treated with 150 mg kg^−1^ 5-FU as described above. One hour after the last 5-FU treatment, three mice from each group (n = 3) were inoculated i.t. with 200 μl (4 injections of approximately 50 μl each) of SFV1/EnhLuc particle-containing stocks (6 × 10^9^ v.p. ml^−1^). 18 hours after the virus administration, 4 T1 tumors were isolated and frozen in liquid nitrogen. Frozen tumors were manually homogenized with homogenization hammer and tissue powders were resuspended in 500 μl of PBS. To provide better tumors homogenization, two freeze-thaw cycles were performed. After homogenization, samples were centrifuged for 10 min at 5000 × g and the protein concentration was equalized in all tissue lysates using the BCA Protein Assay Kit (Pierce™ BCA Protein Assay Kit, Thermo Scientific, UK). Expression of IFN-alpha in 4 T1 lisates was determined using ELISA Kit for Interferon Alpha (Uscn Life Science Inc., China), according provided protocol. The obtained data (pg/ml) were expressed in % relative to lysates non-treated with both the 5-FU and the virus.

### Statistical analysis

The cell viability and RLU results are presented as the means ± standard error of 3 independent experiments. The statistical analysis of the results was performed using Microsoft Excel and Statistica7 (StatSoft, Tulsa, OK, USA). Statistically significant differences were determined using Student’s t-test (P < 0.05).

## Results

### Transduction efficiency and cytotoxicity of alphaviral vectors in 4 T1 cells

**Figure 1:**
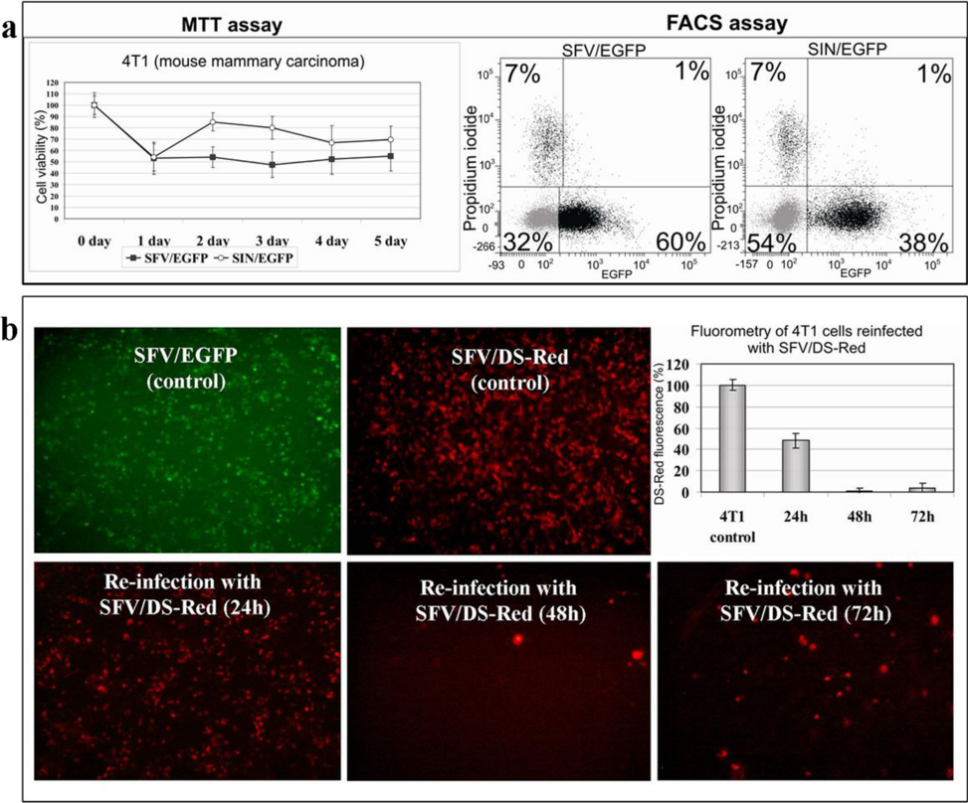
**Transduction efficiency and cytotoxicity of SFV and SIN alphaviral vectors in 4 T1 cells. (a)** 4 T1 cells were infected with SFV and SIN particles encoding EGFP. At 24 h post-infection, the cells were harvested, stained with PI and subjected to dual FACS analysis. The x-axis and the y-axis represent E*GFP* and *PI* fluorescence, respectively. The percentage of living/dead cells and EGFP-positive/negative cells is indicated on the plot. The FACS data shown are from representative experiments (n = 3). The diagram on the left (MTT assay) demonstrates the cytotoxic effects of SFV and SIN infection. An MTT cell viability assay was performed every day for 5 days post-infection. The results are presented as the percentage of viable cells relative to the control (uninfected cells). The error bars indicate the standard error of 3 independent experiments. **(b)** Repeated infection of 4 T1 cells. The cells were infected with SFV expressing EGFP (pictures show green fluorescence) and then re-infected 24, 48 and 72 h later with SFV expressing DS-Red (pictures show red fluorescence). Fluorometry of DS-Red fluorescence was performed 1 day after each re-infection. The diagrams represent the percentage of fluorescence units in re-infected cells relative to control cells (100%), which were primarily infected with only SFV/DS-Red. The error bars indicate the standard error of three experiments.

To select the most efficient cytotoxic alphaviral vector for 4 T1 mouse mammary carcinoma cells, we compared cell survival and transduction efficiency for two commonly used vectors based on SFV and SIN replicons. 4 T1 cells were infected with equal amounts of recombinant particles (multiplicity of infection, MOI = 10) encoding the EGFP gene. FACS analysis of EGFP-positive cells was performed at 24 h post-infection. As shown in Figure 1a (FACS assay), the SFV vector yielded a higher proportion of EGFP-positive cells (60%) compared with the SIN vector (38%).The percentage of EGFP-positive cells measured via FACS indicates the transduction efficiency and the ability of the vector to express the gene of interest. However, alphaviral vectors may provoke cytopathic effects without generating observable transgene expression. This discrepancy is due to the strong induction of rapid apoptosis, which prevents the accumulation of the recombinant product within the cell. To evaluate the immediate (24 h after infection) cytotoxic effects of alphaviral infection, 4 T1 cells were stained with propidium iodide (PI), a membrane-impermeable fluorescent dye that is generally excluded from viable cells. The percentage of PI-positive (dead) cells measured by FACS was similar for the SFV and SIN vectors (7%) (Figure 1a). Nevertheless, the SFV vector provoked a stronger inhibition of cell proliferation than the SIN vector in 4 T1 cells, as demonstrated by the MTT cell viability assays performed over the 5 days following infection (Figure 1a). Despite the strong cytotoxic effect of the SFV vector, the 4 T1 cell culture (in contrast with other highly infectable cancer cell lines, e.g., Huh-7, PA1, H2-35, not shown) survived infection at present conditions, and cell proliferation was completely restored within 8–10 days.

Repeated infections were next tested as a means of enhancing the infectivity and cytotoxicity of the alphavirus. Remarkably, repeated infection of surviving cell culture with the same or a different alphaviral vector (SFV or SIN, respectively) did not produce a significant enhancement of transgene production or prolongation of cytotoxicity. As shown in Figure 1b, the 4 T1 cell culture infected with SFV/EGFP were less susceptible to repeated infection with SFV/DS-Red particles encoding the DS-Red fluorescent protein [34]. Only a very small number of EGFP-negative cells (which did not express the transgene after the first infection) were able to express the DS-Red gene, indicating that the cells could not be doubly infected by both alphaviruses. Similar results were obtained with the SIN vector and with other combinations of SFV/SIN and SIN/SFV reinfection (not shown). Moreover, an MTT cell viability analysis did not reveal a difference in the cell proliferation patterns of singly and doubly-infected cells (not shown). We conclude that the repeated application of alphaviral vectors is not an efficient strategy to achieve complete inhibition of cancer cell proliferation. This effect may be attributable to the overall cellular protein synthesis down regulation [11] and strong induction of an anti-viral response [36,37] that makes the repeated application of the vector inefficient.

The SFV vector was selected for further cytotoxicity analysis in combination with 5-FU.

### Combined treatment of 4 T1 cells with SFV and 5-FU

The low efficiency of oncolytic virotherapy in preclinical studies might be associated with anti-vector immunity or the resistance of tumors to repeated infections. Recently, multiple strategies involving the combination of oncolytic vectors with classic cytotoxic drugs have proven to be advantageous for certain types of cancer (for review, see Wennier et al. 2012) [1]. Here, we analyzed whether the combination of the SFV alphaviral vector and 5-FU exerts a synergetic effect on cancer cell proliferation.

**Figure 2:**
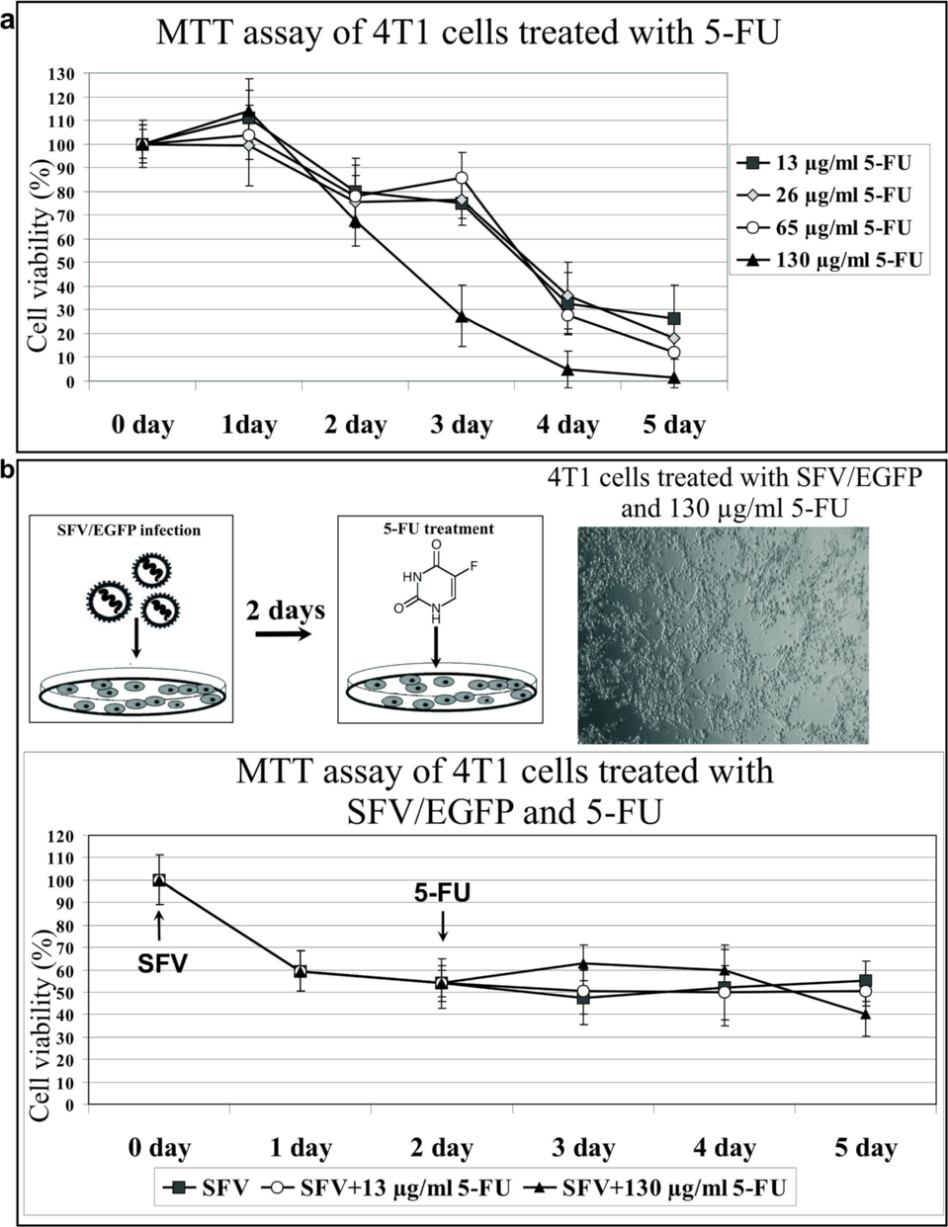
**Evaluation of 4 T1 cell proliferation after 5-FU treatment and in combination with SFV infection. (a)** 5-FU treatment. 4 T1 cells were grown in cell culture medium (24-well plates) containing the indicated concentrations of 5-FU. The MTT cell viability assay was performed every day for 5 days. The diagram shows the cytotoxic effect of 5-FU on 4 T1 cells as the percentage of viable cells relative to the control (untreated cells). **(b)** Schematic representation of the combined treatment with SFV and 5-FU. The cells were infected with SFV/EGFP particles, and the medium was replaced 2 days later with medium containing 5-FU. The MTT cell viability assay was performed every day for 5 days. The arrows designate the day of infection (SFV) and the beginning of the drug treatment (5-FU). The diagram shows the cytotoxic effect of 5-FU following SFV infection as the percentage of viable cells relative to the control (untreated cells). The error bars indicate the standard error of 3 independent experiments. The microscopy image shows a 4 T1 cell monolayer at day 5 after treatment with SFV and the highest concentration of 5-FU.

To analyze the cytotoxic effect of 5-FU on 4 T1 cells, cell monolayers were exposed to different concentrations of 5-FU for 5 days (Figure 2a). After 5 days of incubation, high concentrations of 5-FU (65 and 130 μg ml^−1^) resulted in complete inhibition of cell proliferation on days 5 and 4, respectively. Cells incubated with a low concentration of 5-FU (13 μg ml^−1^) displayed approximately 25% viability on day 5, but further incubation did not lead to complete cell death under these conditions. For the combined treatment, the highest (130 μg ml^−1^) and the lowest (13 μg ml^−1^) 5-FU doses were tested.

The notion that recombinant alphaviruses expressing, e.g., anti-tumor genes and/or inducing anti-tumor immune responses must be applied prior to chemical drug treatment is rational. Therefore, we first tested whether 5-FU could inhibit the proliferation of cells previously infected with SFV. As shown in Figure 2b, 4 T1 cells were infected with SFV/EGFP 2 days prior to treatment with 5-FU. The kinetics of 4 T1 cell proliferation in the combined treatment approach (SFV plus 5-FU) was similar to those of infected 4 T1 cells. The SFV infection of 4 T1 cells alone resulted in 55% of cell viability on day 5 after infection (Figure 1a, MTT-test, SFV). In the case of combined treatment, the cell viability was not significantly changed and resulted in 50% and 40% viability after treatment with 13 μg and 130 μg of 5-FU on day 5, respectively (Figure 2b). Therefore, the application of 5-FU after SFV did not significantly influence the survival of the 4 T1 cell culture, even at the high drug dose (130 μg ml^−1^), providing the evidence for infected cell culture resistance to further treatment with cytotoxic agent.

**Figure 3:**
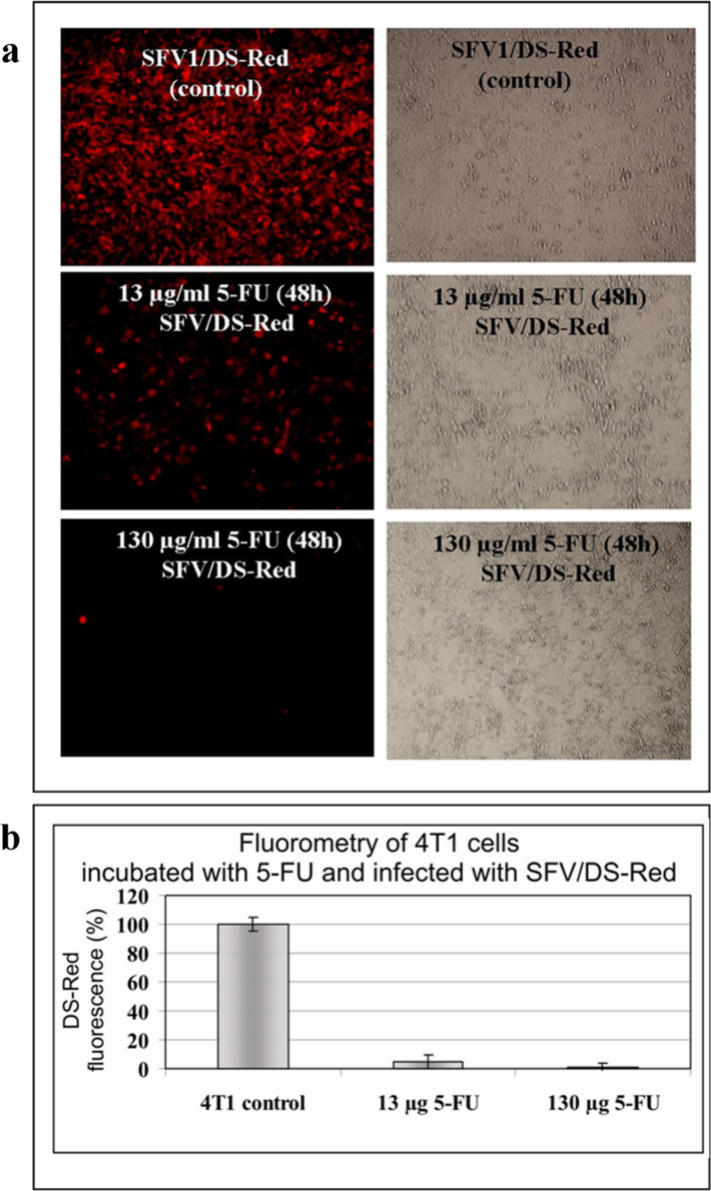
**Inhibition of** S**FV/DS-Red infection in 4 T1 cells pretreated with 5-FU.** 4 T1 cells were treated with high (130 μg ml^−1^) or low (13 μg ml^−1^) concentrations of 5-FU for 2 days, then infected with SFV/DS-Red particles. **(a)** Fluorescence and phase contrast microscopy pictures. **(b)** Fluorometric measurement of DS-Red fluorescence in infected cells at 24 h post-infection. The diagram shows the percentage of fluorescence units measured in the cells pretreated with 5-FU (13 μg ml^−1^ or 130 μg ml^−1^) and then infected with SFV/DS-Red relative to 4 T1 control cells (100%) that were only infected with SFV/DS-Red. The error bars indicate the standard error of three independent experiments.

Short pretreatment of cancer cells with 5-FU has recently been shown to significantly enhance the infectivity of adenoviruses [30,38]. To investigate the effect of 5-FU on alphavirus infection, 4 T1 cells were pretreated with high (130 μg ml^−1^) and low (13 μg ml^−1^) concentrations of 5-FU for 2 days and then infected with SFV/DS-Red. As shown in Figure 3, preincubation of cells with 5-FU almost completely inhibited alphaviral infection. Moreover, in contrast to the adenoviral vector, a short (2 h) pretreatment of 4 T1 cells with a low dose of 5-FU (13 μg ml^−1^) slightly inhibited alphaviral infection, with a total decrease in fluorescence of approximately 10-15% compared with infected cells not treated with 5-FU (not shown). Lower 5-FU concentrations (below 13 μg ml^−1^) had no significant effect on alphaviral infectivity in 4 T1 cells (not shown).

**Figure 4:**
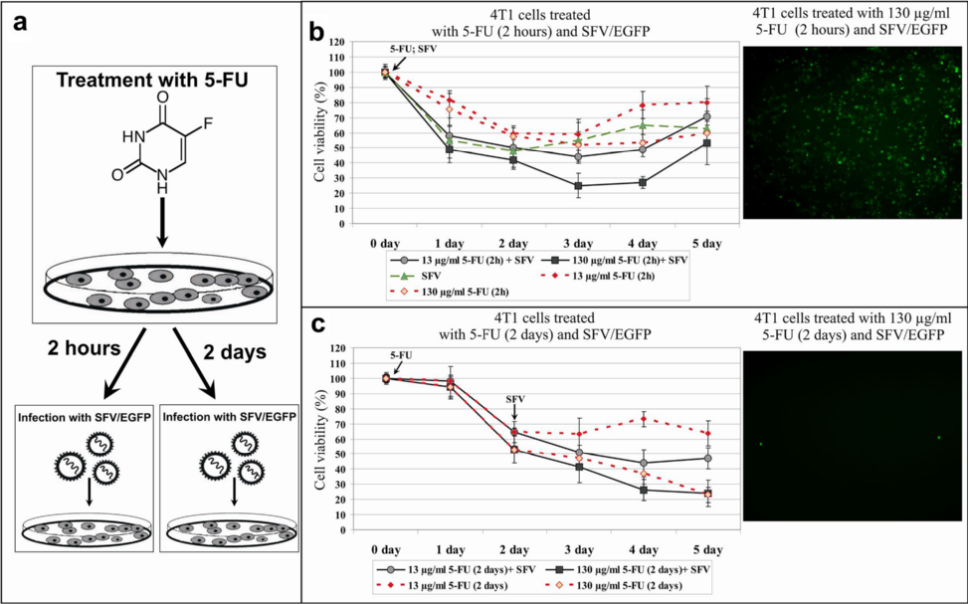
**Evaluation of cytotoxicity in 4 T1 cells treated with 5-FU and then infected with SFV. (a)** Schematic representation of the experiment. 4 T1 cells were pretreated with high (130 μg ml^−1^) or low (13 μg ml^−1^) doses of 5-FU for 2 h or 2 days and then infected with SFV/EGFP particles. **(b)** 4 T1 cells treated with 5-FU for 2 h and infected with SFV/EGFP particles (solid lines). The dotted lines (red) show the controls: cells treated with 5-FU for 2 h and then incubated in complete medium for 5 days. The dashed line (green) shows the cells infected with SFV/EGFP particles. **(c)** 4 T1 cells treated with 5-FU for 2 days and infected with SFV/EGFP particles (solid lines). The dotted lines (red) show the controls: cells treated with 5-FU for 2 days (day 0–2) and then incubated in complete medium for further three days. An MTT cell viability assay was performed every day for 5 days. The diagrams show the cytotoxic effects of 5-FU and SFV/EGFP, which are expressed as the percentage of viable cells relative to the untreated cells. Arrows indicate the beginning of drug treatment (5-FU) and the day of infection (SFV). Error bars show the standard error of three experiments. Fluorescent images demonstrate the efficiency of SFV/EGFP expression on the day after infection of 5-FU pretreated 4 T1 cells.

To measure the inhibition of cell proliferation produced by the combined treatment, 5-FU-pretreated 4 T1 cells were infected with SFV and subjected to cell viability analysis over a period of 5 days (Figure 4). Pretreatment of 4 T1 cells for 2 h with a high dose of 5-FU (130 μg ml^−1^) followed by infection with SFV did not significantly impair cell proliferation compared with 4 T1 cells that were only infected with SFV (Figure 4b). On day 5, the cell viability was approximately 52%. In a similar way, application of a low dose of 5-FU (13 μg ml^−1^) for 2 h did not provoke a significant enhancement of cytotoxic effect of SFV (70% on day 5) compared to the SFV infection alone (60% on day 5), indicating the absence of synergy between 5-FU and SFV. Furthermore, prolonged incubation with 5-FU (for 2 days) also did not produce a significant difference in infected cell proliferation at either dose tested, comparing to uninfected cells under similar conditions (Figure 4c). The cells that were pretreated with a low dose of 5-FU began to resume cell division (49% cell viability) by day 5, whereas the cells treated with a high dose reached 24% cell viability, similar to the controls: cells that were treated with 5-FU but not infected with SFV (64% and 23%, respectively). Therefore, the treatment strategy, in which 5-FU was used prior to virus infection, strongly inhibited SFV expression and did not produce synergistic cytotoxic effect in 4 T1 cells.

### The effect of 5-FU treatment on SFV expression in 4 T1 tumor-bearing mice

**Figure 5:**
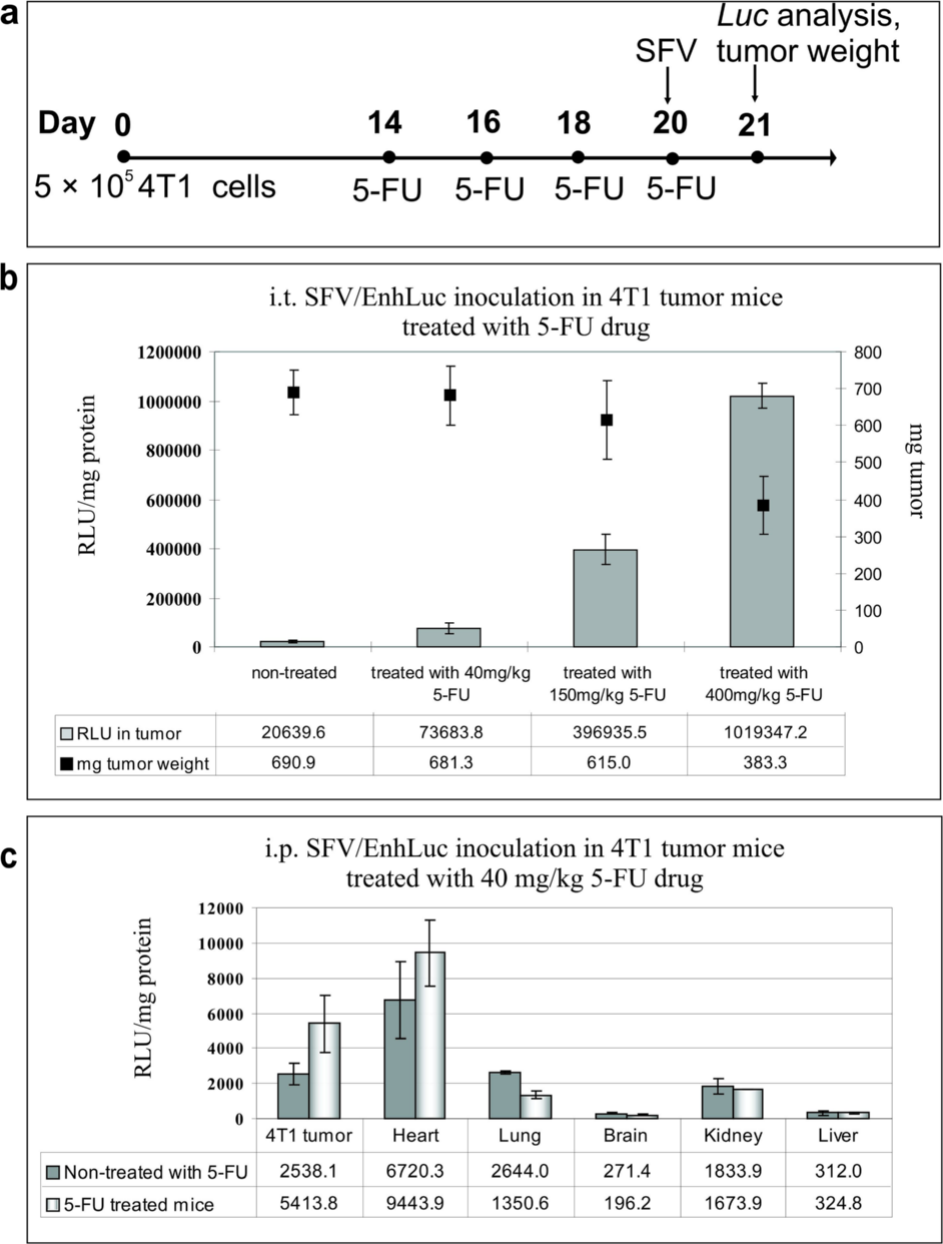
**SFV expression in 4 T1 tumor-bearing mice treated with 5-FU. (a)** Experiment design: Balb/c mice (n = 5 in each group) were subcutaneously inoculated with 4 T1 cells; beginning on day 14, the mice were treated four times with 5-FU, every other day (40 mg kg^−1^, 150 mg kg^−1^ or 400 mg kg^−1^). On day 20, after the last 5-FU administration, the mice were i.t. or i.p. inoculated with SFV/EnhLuc virus particles. Tumor weight and Luc gene expression were measured 24 h after viral inoculation. **(b)** Intratumoral Luc gene expression after i.t injection of SFV/EnhLuc virus particles in 5-FU-treated mice. Luciferase activity was measured in tumor homogenates 24 h after virus inoculation. Tumor weights were measured prior to homogenization (scale on the right). **(c)** SFV/EnhLuc virus biodistribution in 4 T1 tumor-bearing mice treated with 40 mg kg^−1^ 5-FU. Luciferase activity was measured in tumor and organ homogenates 24 h after i.p. virus inoculation. The graphs present the RLUs per mg protein in each organ or tumor (see Methods section). The results are presented as the means ± s.e. The average RLU values and tumor weights are indicated in the tables. RLU, relative light unit.

To investigate the efficiency of SFV-driven transgene expression after 5-FU chemotherapy, 4 T1 tumor-bearing mice were perorally (p.o.) treated with 5-FU and then inoculated with SFV/EnhLuc by intratumoral (i.t.) injection of 3 × 10^8^ virus particles encoding firefly luciferase. The mice were treated with different doses of 5-FU 4 times, every other day (Figure 5a). The lower dose (40 mg kg^−1^) resulted in no visible toxic effects or any significant tumor inhibition; this dose is therefore considered asymptomatic. The medium dose (150 mg kg^−1^) produced a minimal tumor size reduction and medium toxicity (loss of appetite). The high dose (400 mg kg^−1^), by contrast, yielded significant tumor inhibition and strong side effects (watery diarrhea, weight loss, hunched posture). After the last 5-FU treatment (1 h later), the mice were i.t. inoculated with SFV/EnhLuc virus particles, and Luc gene expression was measured 24 h later via luminometry on tumor lysates. The highest luciferase activity was detected in the tumors of mice treated with the highest dose of 5-FU (400 mg kg^−1^) (Figure 5b), with increases in transgene production of approximately 50-fold compared with mice not treated with 5-FU and approximately 14-fold compared with the low dose treatment (40 mg kg^−1^). Remarkably, this asymptomatic low dose also produced a statistically significant 3.6-fold increase in luciferase activity (p < 0.05).

Because the low dose improved transgene expression and had no signs of toxicity, this dose was used to evaluate the tumor targeting and biodistribution of SFV particles upon intraperitoneal (i.p. 1.8 × 10^9^ v.p.) administration in combination with 5-FU. As presented in Figure 5c, the highest levels of Luc gene expression were detected in the tumors and hearts of mice treated with 40 mg kg^−1^ 5-FU. Although significantly lower total Luc expression was observed with i.p. inoculation compared with the i.t. route, the Luc level in the tumors was still 2.1-fold higher (p < 0.05) in i.p. inoculated mice relative to 5-FU untreated mice. Among the other organs, only the heart showed an increase in Luc expression after 5-FU treatment (1.4-fold; not significant). Remarkably, there were no significant changes in vector biodistribution observed in the case of i.t. administration (not shown). The i.t. inoculation provided no further distribution of the vector to organs in both 5-FU treated and untreated mice, confirming therefore the enhancement of vector expression specifically in tumor of 5-FU treated animals.

## Discussion

One strategy to enhance cancer virotherapy is to apply viral vectors in combination with standard and well-studied chemical drugs to promote synergistic actions and potentially lead to effective therapy outcomes. Classic alphaviral vectors based on SFV and SIN replicons have been used for *in vitro* and *in vivo* cancer gene therapy experiments and have shown promising results in different cancer models [39,40]. Nevertheless, the problems of tumor recovery and the inefficiency of repeated vector administration remain to be solved. In this study, we explored the efficiency of SFV-mediated gene transfer in combination with 5-FU and the possibility of a synergistic cytotoxic effect of the combined treatment in the highly proliferative 4 T1 mouse breast cancer model.

5-FU is an antitumor drug typically included in breast carcinoma chemotherapeutic regimens [41,42]. The cytotoxic effect of 5-FU occurs through the inhibition of the synthesis and functioning of DNA and RNA. Although the general mechanism of 5-FU action as an anti-metabolite has been investigated [43], little is known about the intracellular molecular changes that lead to apoptosis in the presence of 5-FU. Protein kinase R (PKR) has been shown to be a molecular target of 5-FU-induced apoptosis [44], suggesting that 5-FU might induce apoptosis via a mechanism similar to that of alphaviruses: the double-stranded RNA intermediates made during alphavirus genome/subgenome replication also activate PKR, which contributes to the inhibition of protein synthesis [45]. PKR has also been shown to play an important role in the induction of apoptosis by other drugs, such as doxorubicin and etoposide [46,47], which have been successfully used in combination with other viruses [48,49]. Therefore, the combined treatment with alphavirus and 5-FU presented herein could potentially produce a synergistic effect due to the targeting of similar pathways that may work together to enhance cytotoxicity in cancer cells. Nevertheless, this combined treatment showed poor efficiency in 4 T1 cells *in vitro*. Neither SFV infection with subsequent 5-FU treatment (Figure 2b) nor the opposite strategy of pretreatment with 5-FU and later infection with SFV (Figure 4) produced a more efficient inhibition of cell proliferation compared with SFV or 5-FU alone (Figures 1a and 2a). Moreover, pretreatment of cells with 5-FU significantly inhibited SFV infection and transgene expression (Figure 3).

The basis for the resistance of the surviving cell population to high 5-FU doses and SFV infection in the combined treatment remains unclear. Cabrele et al. [4] and others demonstrated stimulation of adenoviral vector infection via 2 h of low-dose pretreatment with 5-FU in human colon carcinoma cell lines. In contrast to adenoviruses, RNA containing alphaviruses replicate their genome in the cytoplasm. The extremely efficient alphaviral RNA replication is regulated by the virus-encoded replicase complex and the specific secondary structure of the RNA genome [50]. As previously described, incorporation of 5-FU metabolites into RNA may change RNA structure and/or affect tRNA and rRNA function [43]. It is thus possible that a similar incorporation of 5-FU metabolites into alphaviral genomic and subgenomic RNAs may likewise alter RNA secondary structure and inhibit its replication and translation. The presence of 5-FU and its metabolites could also inhibit the viral replicase in a similar manner to that observed in the inhibition of the active center of thymidylate synthetase by 5-fluorodeoxyuridine monophosphate [51]. We conclude that this combined treatment produces no synergy in the induction of apoptosis but rather inhibits alphaviral replication and transgene production.

Several oncolytic viruses have been applied in combined treatments in mouse tumor models [1]. However, less is known about the efficiency of infection or the kinetics of virus persistence under combined treatment in mice because most studies focused on the significant therapeutic effects and tumor growth inhibition. The fact that multiple different combinations of viruses (enveloped, unenveloped, dsDNA, RNA, ssDNA) and cytotoxic chemical drugs (antimetabolites, antibiotics) all produce synergistic therapeutic effects implies a common non-specific mechanism underlying such a benefit. Here, we observed a significant enhancement of intratumoral SFV-mediated transgene expression in mice treated with 5-FU (Figure 5). The low dose (suboptimal) of 5-FU provoked a 3.6-fold increase in Luc gene expression, whereas the high dose (400 mg kg^−1^, which is close to the maximum-tolerated dose of chemotherapy regimens) yielded a 50-fold increase. This positive correlation between 5-FU dose and the level of Luc expression contradicts the *in vitro* results; however, this correlation is in line with the promising results obtained using other viruses in combination with 5-FU in mouse models [52-54].

**Figure 6:**
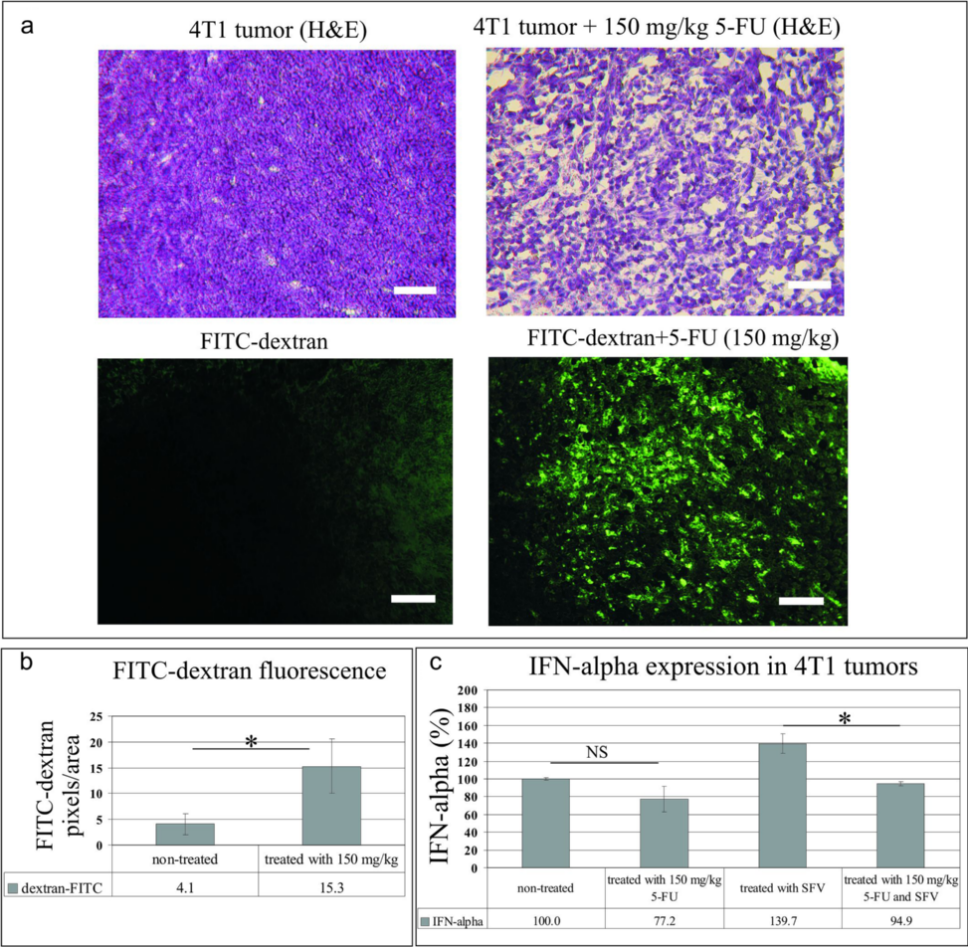
**Evaluation of vascular permeability and intratumoral level of IFN-alpha in 4 T1 tumor-bearing mice treated with 5-FU. (a)** Fluorescent microscopy of 4 T1 tumor cryosections demonstrating a FITC-dextran 2000 kDa accumulation in untreated (left) and 5-FU treated (right) tumors. FITC-dextran 2000 kDa (green) was intravenously-administrated and 2 h later tumors were processed as described in methods. FITC, fluorescein isothiocyanate. H&E, hematoxylin and eosin staining of cryosections. The 5-FU exhibited morphological changes in tumor sections, microvessel density was significantly lower than that in the untreated control, large patchy necrosis was visualized. **(b)** The diagram shows the data from three tumors, evaluating ten sections per tumor. **(c)** IFN-alpha response to intratumoral SFV/EnhLuc injections in tumor-bearing mice treated and untreated with 5-FU. The IFN-alpha level is expressed in % relative to tumors non-treated with 5-FU and virus. Two groups of mice (n = 6) were treated and non-treated with 5-FU, respectively, then a half of each group of mice was subjected to intratumoral injections of SFV/EnhLuc. The IFN-alpha response was measured in tumor homogenates after 18 h of virus injection. NS – non-significant differences; * - significant differences (p < 0.05), mean ± SD; bar 200 μm.

5-FU is widely distributed to all tissues, including sites of active cell proliferation [55]. In addition to the tumor, the primary target cells are endothelial cells in blood vessels. Therefore, 5-FU treatment leads to massive cell death in places with high vascularization (including the tumor), which may increase tissue permeability to macromolecules and viruses in particular. The high level of SFV expression observed following 5-FU treatment might be explained by the enhanced permeability and retention (EPR) effect [56,57], which leads to passive and preferential accumulation and more efficient intratumoral distribution of the virus at sites of enhanced vascular permeability. To compare tumor vascular leakage in mice treated and untreatedwith 5-FU, we used fluorescein isothiocianate-conjugated dextran 2000 kDa (FITC-dextran 2000), a polysaccharide with a high molecular mass that is used as a model of permeability and retention for macromolecular structures such as nanoparticles, liposomes and viruses [58]. FITC-dextran 2000 was injected via the tail vein in 4 T1 tumor bearing mice and 2 h later the tumor cryosections were subjected to fluorescence analysis. As shown in Figure 6a and b, the distribution intensity of FITC-dextran 2000 within the tumor was significantly higher in 5-FU treated mice (150 mg kg^−1^) comparing to the untreated control. This observation supports the idea that 5-FU treatment elevates tumor vascular permeability of macromolecular structures that might lead to enhanced virus distribution and high level of transgene production in 5-FU treated animals. The concept of enhanced virus intratumoral distribution after drug treatment is also supported by the results of Tseng et al. [59], who demonstrated a significant enhancement in tumor vascular permeability and oncolytic Sindbis vector targeting following chemotherapy. Notably, those authors also did not observe a positive effect of the drug treatment (paclitaxel) on virus infection and replication *in vitro*.

Besides the changes in tumor vascular permeability mediated by 5-FU treatment, an antiviral immune response has to be considered as a factor which affects the infection. At the early step of infection alphaviruses are sensitive to type I IFN production [60,61]. We have examined the intratumoral level of IFN-alpha in 5-FU treated and untreated tumor bearing mice as a response to i.t. administrated SFV (Figure 6c). The results indicate a significant inhibition of IFN-alpha antiviral response in 5-FU treated tumors, evidencing the innate immunity inhibition by 5-FU that at the same time might lead to enhanced virus replication.

Therefore, we propose that pretreatment with a cytotoxic drug may enhance the efficiency of alphaviral-mediated transgene delivery through the EPR effect and the inhibition of antiviral IFN-alpha response. Here we have demonstrated a significant 3.6-50.0 fold increase in Luc transgene expression that can be regulated by 5-FU dose. Although we did not observe any differences in tumor growth and survival rates (not shown) between the groups of animals treated with 5-FU and treated with combination of 5-FU and SFV/EnhLuc, the observed enhancement of intratumoral virus expression mediated by 5-FU pretreatment has a potential to advance the alphavirus-driven transgene delivery field. The insertion of proinflammatory transgenes into the vector instead of reporter *luc* gene could be promising for further optimization of SFV-based virotherapy of cancer to enhance the effect of chemotherapy and to prevent tumor recurrence and metastasis.

## Conclusions

**Figure 7:**
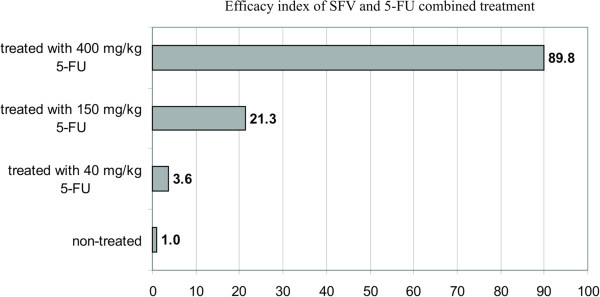
**The efficacy index of SFV and 5-FU combined treatment of 4 T1 tumor-bearing mice.** The mice were treated with different doses of 5-FU and then i.t. inoculated with SFV/EnhLuc virus particles. The efficacy index reflects the correlation of tumor growth inhibition with the level of SFV expression in 5-FU treated mice. The calculations are described in the Methods section.

In this study, we describe the enhanced intratumoral expression of a replication-deficient SFV vector following 5-FU treatment in the 4 T1 mouse mammary tumor model. To illustrate the efficacy of the combined treatment, we introduced “the efficacy index”, which revealed a decrease in tumor weight upon 5-FU treatment that was correlated with an increase in SFV expression. As presented in Figure 7, the highest efficacy index (89.8) was observed with the high 400 mg kg^−1^ 5-FU treatment, which provoked a significant inhibition of tumor growth and the most efficient intratumoral SFV expression. The application of a subtherapeutic dose (40 mg kg^−1^) of 5-FU also led to a 3.6-fold enhancement of SFV expression upon i.t. vector administration. Moreover, 5-FU treatment did not change the distribution of SFV upon i.p. inoculation, allowing preferential vector expression in the tumor and heart and leading to a 2.1-fold increase in intratumoral SFV expression.

Although the combined treatment did not show a synergistic anti-proliferative effect *in vitro* due to the strong inhibition of SFV replication by 5-FU, the significant increase observed in intratumoral SFV expression (even at a low drug dose) might enhance the transgene delivery of alphaviral vectors and their general therapeutic potential.

## Competing interest

The authors declare no conflict of interest.

## Authors’ contributions

AZ conducted the study, participated in its design and data interpretation and wrote the manuscript; JV carried out the *in vitro* and *in vivo* studies, performed the calculations, produce the figures; DZ carried out the fluorometric assay; DS carried out experiments with animals; AS performed FACS analysis; AP helped with 5-FU application; TK participated in coordination of the study and data analysis. All authors read and approved the final manuscript.

## Pre-publication history

The pre-publication history for this paper can be accessed here:

http://www.biomedcentral.com/1471-2407/14/460/prepub
